# Decreased Neutrophil Apoptosis in Quiescent ANCA-Associated Systemic Vasculitis

**DOI:** 10.1371/journal.pone.0032439

**Published:** 2012-03-05

**Authors:** Mohamed Abdgawad, Åsa Pettersson, Lena Gunnarsson, Anders A. Bengtsson, Pierre Geborek, Lars Nilsson, Mårten Segelmark, Thomas Hellmark

**Affiliations:** 1 Department of Nephrology, Clinical Sciences, Lund University, Lund, Sweden; 2 Department of Rheumatology, Clinical Sciences, Lund University, Lund, Sweden; 3 Department of Haematology, Laboratory Medicine, Lund University, Lund, Sweden; 4 Departemnt of Health and Medicine, Linköping University, Linköping, Sweden; Wayne State University, United States of America

## Abstract

**Background:**

ANCA-Associated Systemic Vasculitis (AASV) is characterized by leukocytoclasis, accumulation of unscavenged apoptotic and necrotic neutrophils in perivascular tissues. Dysregulation of neutrophil cell death may contribute directly to the pathogenesis of AASV.

**Methods:**

Neutrophils from Healthy Blood Donors (HBD), patients with AASV most in complete remission, Polycythemia Vera (PV), Systemic Lupus Erythematosus (SLE), Rheumatoid Arthritis (RA) and renal transplant recipients (TP) were incubated *in vitro*, and the rate of spontaneous apoptosis was measured by FACS. Plasma levels of cytokines and sFAS were measured with cytometric bead array and ELISA. Expression of pro/anti-apoptotic factors, transcription factors C/EBP-α, C/EBP-β and PU.1 and inhibitors of survival/JAK2-pathway were measured by real-time-PCR.

**Results:**

AASV, PV and RA neutrophils had a significantly lower rate of apoptosis compared to HBD neutrophils (AASV 50±14% vs. HBD 64±11%, p<0.0001). In RA but not in AASV and PV, low apoptosis rate correlated with increased plasma levels of GM-CSF and high mRNA levels of anti-apoptotic factors Bcl-2A1 and Mcl-1. AASV patients had normal levels of G-CSF, GM-CSF and IL-3. Both C/EBP-α, C/EBP-β were significantly higher in neutrophils from AASV patients than HBD. Levels of sFAS were significantly higher in AASV compared to HBD.

**Conclusion:**

Neutrophil apoptosis rates *in vitro* are decreased in AASV, RA and PV but mechanisms seem to differ. Increased mRNA levels of granulopoiesis-associated transcription factors and increased levels of sFAS in plasma were observed in AASV. Additional studies are required to define the mechanisms behind the decreased apoptosis rates, and possible connections with accumulation of dying neutrophils in regions of vascular lesions in AASV patients.

## Introduction

AASV is characterized by leukocytoclasis, infiltration and accumulation of unscavenged apoptotic or necrotic neutrophils in perivascular tissues and fibrinoid necrosis of the vessel walls [1] [2]. Activated, apoptotic and necrotic neutrophils are seen in histological samples from patients with Granulomatosis with polyangiitis (GPA) with respiratory disease [3]. Histological evidence suggests that neutrophil apoptosis may play a central role in the pathogenesis of AASV and production of ANCA (Anti-Neutrophil Cytoplasmic Antibodies) [4] [5]. Injection of brown Norway rats with syngenic apoptotic neutrophils was shown to induce ANCA but not AASV, suggesting that additional factors are required for disease pathogenesis [6].

Higher levels of plasma PR3 have been reported in patients with quiescent AASV (in remission) compared to Healthy Blood Donors (HBD) [7] [8]. Furthermore, membrane PR3-positive (mPR3**^+^**) neutrophils are more abundant in individuals with quiescent AASV indicating that PR3 plays an active role in the pathogenesis of AASV and not just a marker of inflammation. It has been shown that PR3 can trigger cultured endothelial cell apoptosis; however, the mechanism was not defined [9]. PR3 activates procaspase-3 into a specific 22-kDa fragment, localized to the plasma membrane-enriched compartment and segregated from its target cytosolic proteins that promote apoptosis, thus causing activation but not apoptosis [10]. Vong et al showed that recombinant PR3 or the membrane fraction of cells stably-transfected with PR3 can cleave Annexin-A1 (AnxA1), suggesting that AnxA1 may be a physiologically relevant substrate for PR3 [11]; AnxA1 was recently recognized as an important inducer or promotor of neutrophil apoptosis. Harper et al reported faster apoptosis in neutrophils from patients with active vasculitis compared to neutrophils from patients with quiescent vasculitis or from HBD; neutrophils from patients with active vasculitis also had higher levels of mPR3 and superoxide production [12].

PR3 can be mobilized to the plasma membrane in the absence of prior neutrophil priming and independent of degranulation during the apoptotic process [13]. Kantari et al showed that Phospho-Lipid scramblase-1 (PLSCR-1) interacts with PR3 and promotes its translocation to the plasma membrane in a flip-flop manner during apoptosis [14]. However, the level of mPR3 is similar in apoptotic and non-apoptotic primed neutrophils, implying that the mPR3 on apoptotic neutrophils may be a result of minor trauma during neutrophil isolation [15]. Our group has previously shown that the level of mPR3 on aging neutrophils is slowly decreasing and is not a pre-apoptotic marker [16]. Thus, PR3 seems to be linked to neutrophil apoptosis, though the exact nature of this relationship is not clear. As there is definite evidence for increase PR3 and neutrophil accumulation in AASV, it is likely that neutrophil apoptosis may contribute to disease pathogenesis.

Accumulation of neutrophils in AASV tissues may occur as a result of either an increase in granulopoiesis, defective apoptosis or impaired clearance of apoptotic neutrophils. Based on elevation of PR3 in quiescent AASV and its link to neutrophil apoptosis, we hypothesized that the neutrophil apoptosis in AASV is dysregulated even during remission. In this study, we focus on early events explaining the origin of ANCA by studying patients in remission; our attempt was to confirm abnormality in apoptotic rate in AASV and to elucidate the mechanisms underlying the hypothesized dysregulation. The rates of spontaneous *in vitro* apoptosis were noted in neutrophils from AASV patients most in complete remission or with mild activity and other study populations; these were analyzed in relation to clinical data. The expression of selected genes and proteins involved in neutrophil survival was evaluated in relation to apoptosis.

## Methods

### Patients

During the period between September 2006 and February 2008, 44 AASV patients (most in complete remission or having mild activity) from the Department of Nephrology, Lund University Hospital were recruited into the current study. Patients diagnosed with AASV were classified as GPA or Microscopic Polyangiitis (MPA) using the European Medicines Agency (EMEA) algorithm [17]. The vasculitis activity status of all patients was determined using the Birmingham Vasculitis Activity Score (BVAS) [18]. AASV patients were receiving the following treatments at time of sampling: 21 patients- cytotoxic drugs and steroids; 10 patients- cytotoxic drugs; 5 patients- steroids; 8 patients- no treatment (Table 1). None of the patients had received biological treatment.

**Table 1:** Demographic data for the AASV patients and controls.

Variable	GPA	MPA	HBD	PV	TP	SLE	RA
N	31	13	93	17	20	21	21
Age years;	61	64	41	61	51	44	63
median (range)	(18–86)	(37–87)	(21–68)	(37–81)	(29–71)	(22–68)	(32–86)
F/M ratio	15/16	7/6	36/57	7/10	7/13	20/1	13/8
PR3-ANCA	26	1	-	-	-	-	-
MPO-ANCA	3	12	-	-	-	-	-
ANCA negative	2	0	-	-	-	-	-
BVAS 0–1	23	8	-	-	-	-	-
BVAS 2–5	6	5	-	-	-	-	-
BVAS>5	2	0	-	-	-	-	-
DAS; median (IQR)	-	-	-	-	-	-	3.96 (3.1–4.5)
SLEDAI; median (IQR)	-	-	-	-	-	4 (0–8)	-
Prednisolone dose mg/day; median (IQR)	1.25 (0–5)	5 (0–7.5)	-	-	5 (5–7.5)	7 (5–10)	2.5 (0–5)

AASV = ANCA-associated Systemic Vasculitis. GPA = Granulomatosis with polyangiitis. MPA = Microscopic polyangiitis. N = Number of subjects. F = Female. M = Male. HBD = healthy blood donors. PV = Polycythemia Vera. TP = renal transplant recipients. SLE = Systemic Lupus Erythematosus. RA = Rheumatoid Arthritis. BVAS = Birmingham Vasculitis Activity Score. DAS = Disease activity score. SLEDAI = SLE disease activity index. IQR = Interquartile range.

Additional study participants included HBD from the local blood bank, TP recipients from the Department of Nephrology, PV patients from the Department of Haematology, SLE and RA patients from the Department of Rheumatology, all at Lund University Hospital (Table 1). None of the disease controls was treated with biological treatment.

This study was approved by the Regional Ethical Review Board and performed in accordance with the Declaration of Helsinki. Informed signed consent was obtained from all study participants.

### Blood sampling and separation

Leukocytes were isolated by centrifugation on Polymorphprep (Axis- Shield, Oslo, Norway). Plasma band was used to measure levels of different cytokines. The neutrophil band was used to study neutrophil survival, apoptosis and necrosis by FACS and to extract RNA for real time PCR.

### Neutrophil *in vitro* culture and FACS

Isolated neutrophils were cultured in AIM-V medium (neither calf nor human serum was used) and incubated in an incubator with 5% CO2 in humid atmosphere, at 37°C, for 20 h. An aliquot (10^6^ neutrophils) was taken and incubated for 5 min in the dark with 1 µl Annexin-V (marker of apoptosis from Invitrogen, Molecular probes, Oregon, USA) and 10 µl 7-AAD (marker of necrosis from BD-Biosciences, San Jose, CA, USA). Annexin-V was conjugated with Alexa 488 while 7-AAD was conjugated with PE (Phycoerythrin). Neutrophils were then analyzed by flow cytometry using BD FACSCanto II (BD Pharmingen, CA, USA) to report % of apoptotic, necrotic or alive cells after 20 h of *in vitro* culture.

### RNA extraction

Total RNA was extracted with RNeasy Mini kit (Qiagen, VWR International, West Chester, PA, USA) according to the manufacturer's protocol. RNA purity was evaluated by spectrophotometric analysis using NanoDrop (Saveen& Werner, Malmö, Sweden).

### Quantitative PCR assay

cDNA was prepared from total RNA using *Taq*Man Reverse Transcription Kit (Applied Biosystems, Foster City, CA, USA) according to the manufacturer's instructions. Briefly, reverse transcription was performed using random hexamers, MultiScribe reverse transcriptase, RNase inhibitor, dNTPs, 5.5 mM MgCl_2_, reverse transcription buffer, and 200 ng total RNA in a volume of 50 µl. The reaction cycle was 25°/10 min, 48°/30 min and 95°/5 min. Quantitative PCR assays were performed in an ABI PRISM 7000 Sequence Detector (Applied Biosystems, CA, USA) with *Taq*Man Universal Master Mix UNG under standard conditions. Assay on Demand provided a unique combination of forward and reverse primers and fluorescent MGB-probes for each target gene (Bax, Mcl-1, Bcl-2A1, c-IAP2, C/EBP-α, C/EBP-β, PU.1, SHIP-1, SOCS1 and SOCS3). Cyclophilin A expression was used as an internal control for data normalization. Each 25 µl reaction contained the amount of cDNA produced from 10 ng RNA. All reactions were performed in triplicates. Q-PCR data were analyzed using the ΔΔCt method with normalization to Cyclophilin A and standard 2^(−ΔΔ Ct)^ calculations [19].

### Measurement of neutrophil growth factors and soluble FAS in plasma by ELISA and cytometric bead array (CBA)

G-CSF, GM-CSF, IL-3 and soluble Fas (sFAS) were measured in plasma with the Quantikine® ELISA Kit (R&D systems, Abingdon, UK), which was used according to the manufacturer's protocol.

G-CSF, GM-CSF, IL-3, TNF-α, IFN-γ, IL-1β, IL-2, Il-4, IL-6 and IL-8 were simultaneously analyzed in 50 µl plasma by flow cytometry using the BD CBA Human Soluble Protein Flex Set system (BD Pharmingen, CA, USA) according to the manufactures instructions.

### Statistical analysis

For continuous variables, an unpaired t-test was used to measure statistical significance of differences between two groups. Results are presented as mean± SD. For data sets that follow a non-Gaussian distribution, statistical significance was measured using a Mann-Whitney test. Results are presented as median and range or interquartile range (IQR).

One-way ANOVA with Bonferroni's post-test was used to compare data from more than two groups. Correlations were analyzed using Pearson rank test. Spearman rank test was used for non-parametric data. A two-sided p<0.05 was considered to be statistically significant.

## Results

### Neutrophil apoptosis *in vitro*

**Figure 1:**
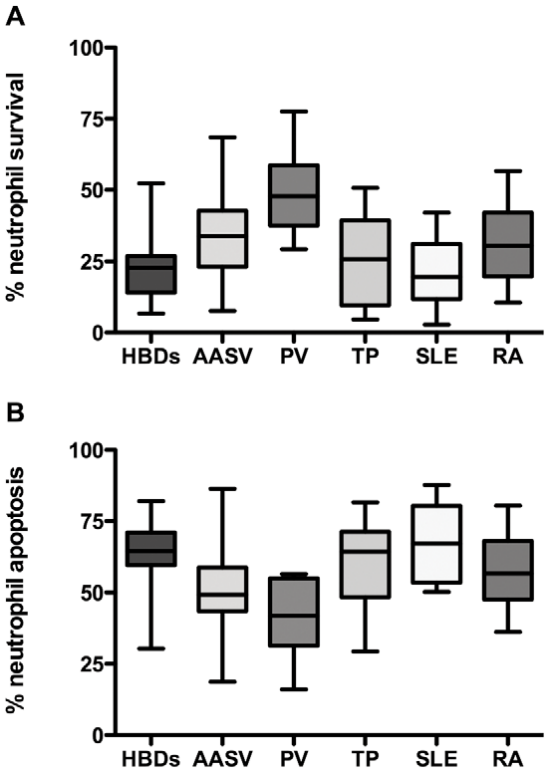
Rate of neutrophil survival and apoptosis. Neutrophils isolated from 60 HBD, 44 AASV patients, 8 PV patients, 18 TP, 21 SLE patients, and 20 RA patients were cultured in vitro in AIM-V medium. The percentage of surviving neutrophils (1a) and apoptotic neutrophils (1b) was measured after 20 hours. % Neutrophil survival = % of annexin-V negative and 7-AAD negative cells after 20 hour culture. % Neutrophil apoptosis = % of annexin-V positive and 7-AAD negative cells after 2o hour culture. HBD = healthy blood donors. AASV = ANCA-Associated Systemic Vasculitis. PV = Polycythemia Vera. TP = renal transplant recipients. SLE = Systemic Lupus Erythematosus. RA = Rheumatoid Arthritis.

Apoptosis, necrosis and survival of neutrophils from 44 patients with AASV, 93 HBD, 20 TP recipients, 17 PV, 21 SLE and 21 RA patients was quantified after 20 h in culture as described in the method section. The results showed a significantly higher rate of survival (mean ±SD 34±13% Vs 23± 9%, p<0.0001; Figure 1a) and lower rate of apoptosis (50±14% Vs 64±11%, p<0.0001; Figure 1b) in AASV neutrophils compared to neutrophils from HBD. Similar results were obtained when examining neutrophils from RA and PV patients, with survival rates of 31±13% and 49±15% (p = 0.015 and p<0.0001), and apoptosis rates of 57±12% and 41±14% (p = 0.027 and p<0.0001). For necrosis there was no significant difference between neutrophils from AASV, PV and RA neutrophils compared to HBD neutrophils (data not shown).

### No correlation between neutrophil apoptosis and clinical data

To ascertain that the reduced rate of apoptosis was not a consequence of gender, age, renal function, general inflammation or treatment, apoptosis rates were correlated with clinical data for the AASV patients. No significant correlations were found with gender (men and women had similar rates: 49±11% vs. 51±15%; p = 0.5, n = 22 for men and n = 22 for women) or age (Spearman r = 0.01, p = 0.9, n = 44). There was no correlation with serum Creatinine concentration (Spearman r = −0.13, p = 0.4, n = 44) or estimated GFR (Spearman r = 0.1, p = 0.5, n = 44). Similar results were observed for patients with GPA and MPA (49±12% Vs 53±16%; p = 0.4; n = 31 for GPA and n = 13 for MPA). There was no correlation with CRP (Spearman r = −0.11, p = 0.5, n = 44). Regarding disease activity, no conclusion could be drawn; since patients in remission (BVAS<2, n = 31) had comparable apoptosis rates as patients with moderate vasculitis activity (BVAS 2–5, n = 11). Patients with high vasculitic activity (BVAS>5, n = 2) had tendency towards lower apoptosis rate (51±14% and 52±9% Vs 28±13%; p = 0.09); this difference was not significant, though the number of patients was too small to draw a definite conclusion.

We could not demonstrate any influence ongoing steroid treatment on the measured rate of apoptosis, as there was no correlation between neutrophil apoptosis and the dose of prednisolone mg/day (Spearman r = −0.11, p = 0.5). We divided the patients into two subgroups; a subgroup treated with steroids and a subgroup without steroids: there was no difference between the two subgroups in regard to neutrophil apoptosis (49±13% Vs 51±15%, P = 0.6). For anti-proliferative drugs the situation was somewhat more complex; the 27 AASV patients who were on treatment tended to have lower apoptosis rates as compared to the 8 patients who were off all kinds immunosuppressive treatment (47±12% Vs 57±15%; p = 0.02), with no obvious difference between drugs. The 10 methotrexate treated patients had a mean rate of 46±11%, compared with 48±12% for 10 patients on azathioprine and 48±13% for 7 patients on mycophenolate mofetil (p = 0.94). In contrast, 11 renal transplant recipients on mycophenolate mofetil had a mean apoptosis rate of 57±16%.

### Plasma factors potentially influencing neutrophil survival in AASV

The plasma levels of G-CSF, GM-CSF, IL-3, TNF-α, IFN-γ, IL-1β, IL-2, IL-4, IL-6 and IL-8 were measured using Cytometric Bead Analysis (CBA), and G-CSF, GM-CSF, IL-3 and sFAS were also quantified using ELISA. For most cytokines examined, there were no significant difference between HBD and AASV. IL-3 was below the detection limit in all tested samples. G-CSF was elevated in plasma samples from 10/44 AASV patients (median, range: 31.7, 20–72 pg/ml) and 4/21 RA patients (26.9, 17–43 pg/ml- Table 2), but there was no correlation between the plasma levels of G-CSF and the rates of neutrophil survival and apoptosis in these patients. Patients were divided into two subgroups according to their G-CSF levels; there was no difference between the two subgroups in their neutrophil survival/apoptosis (Table 2). G-CSF was also detected in 10/93 HBD (28.3, 15–46 pg/ml), which is slightly lower than AASV, but the difference was not significant (P = 0.29).

**Table 2:** Neutrophil survival among patients with high G-CSF levels.

Patients	G-CSF pg/ml	Neutrophil survival %
AASV 1	20.37	33%
AASV 2	22.68	51%
AASV 3	23.69	20%
AASV 4*	27.16	52%
AASV 5	30.76	35%
AASV 6	32.67	40%
AASV 7	33.1	30%
AASV 8	41.5	34%
AASV 9	68.19	34%
AASV 10	72.32	39%
Median of the 10 AASV patients above (range)	31.7 (20–72)	35% (20%–52%)
Median of the 34 remaining AASV patients (range)	UD	33% (8%–68%)
RA 1	17.05	57%
RA 2	22.45	10%
RA 3**	26.98	42%
RA 4	34.55	30%
RA 5***	42.87	31%
Median of the 5 RA patients above (range)	26.9 (17–43)	31% (10%–57%)
Median of the 15 remaining RA patients (range)	UD	30% (12%–48%)

% Neutrophil survival = % of annexin-V negative and 7-AAD negative cells after 20 hour culture. AASV = ANCA-associated Systemic Vasculitis. RA = Rheumatoid Arthritis. GM-CSF = Granulocyte-Colony Stimulating Factor. UD = Undetectable (<2 pg/ml).

*/**/*** Are signals for the common patients between table 2 and 3.

IFN-γ levels were undetectable in plasma of HBD, while 5 AASV patients had elevated levels of IFN-γ in their plasma (median 19 pg/ml, range 8–25 pg/ml).

GM-CSF was <2 pg/ml in the majority of samples, which is within the expected normal range for healthy individuals. GM-CSF was elevated in plasma samples from 4/44 AASV patients (median, range: 484.2, 7.7–3072 pg/ml) and 8/21 RA patients (median, range: 37, 11–178 pg/ml), Table 3. Overall there was no significant correlation between the plasma levels of GM-CSF and the rates of neutrophil survival, apoptosis or necrosis. The 4 out of 44 AASV patients with high GM-CSF showed only marginally higher survival rates and the difference was not statistically significant (38±14% Vs 34±13%, p = 0.5). However the 8 RA patients with elevated GM-CSF levels exhibited a significantly reduced apoptosis rate (51±9% Vs 61±12% apoptosis rate, p = 0.034) combined with a tendency for increased survival (37±10% Vs 27±14%, p = 0.09), Table 3.

**Table 3:** Neutrophil survival among patients with high GM-CSF levels.

Patients	GM-CSF pg/ml	Neutrophil survival %
AASV 1*	7.7	52%
AASV 2	195.71	42%
AASV 3	772.73	41%
AASV 4	3072	18%
Median of the 4 AASV patients above (range)	484 (7.7–3072)	41% (18%–52%)
Median of the 40 remaining AASV patients (range)	UD	33% (8%–68%)
RA 1	11.3	38%
RA 2	27.28	47%
RA 3***	29.15	31%
RA 4	35.41	19%
RA 5	38.48	48%
RA 6	71.89	42%
RA 7**	112.16	42%
RA 8	178.52	30%
Median of the 8 RA patients above (range)	37 (11–178)	40% (19%–48%)
Median of the 12 remaining RA patients (range)	UD	24% (10%–57%)

% Neutrophil survival = % of annexin-V negative and 7-AAD negative cells after 20 hour culture. AASV = ANCA-associated Systemic Vasculitis. RA = Rheumatoid Arthritis. GM-CSF = Granulocyte Macrophage-Colony Stimulating Factor. UD = Undetectable (<2 pg/ml).

*/**/*** Are signals for the common patients between table 2 and 3.

The levels of sFAS were significantly higher in the plasma of AASV patients compared to healthy controls (mean±SD 0.8±0.3 Vs 0.4±0.1 pg/ml, P<0.0001), but there was no correlation between levels and rate of spontaneous apoptosis (Pearson r = 0.01, P = 0.9).

### Apoptosis and proportion of PR3^+^/CD177^+^ neutrophils

In accordance with previous studies we found an increased fraction of neutrophils double positive for membrane PR3 expression and the surface marker CD177 (Mean±SD: 69±19% for AASV, 58±21% for HBD; p = 0.004, n = 52 for AASV and n = 91 for HBD). There was, however, no correlation between the percentage of double-positive neutrophils and the rate of apoptosis in all the groups of all subjects participated in the study (Pearson r = −0.02, p = 0.7, n = 156).

### mRNA expression analysis

The mRNA expression of the following genes was analyzed: anti–apoptotic factors (c-IAP2, Bcl2-A1 and Mcl-1), pro-apoptotic factor (Bax), transcription factors (C/EBP-α, C/EBP-β and PU.1), growth factor receptors (G-CSFR, GM-CSFR alpha, GM-CSFR beta) and suppressors of cytokine signaling (SHIP-1, SOCS1, SOCS3). Quantitative PCR as performed for their respective mRNA transcripts. These were quantified in neutrophils from patients with AASV, HBD, PV, RA and TP.

The results showed slightly higher expression of Bcl-2A1 (Median, range: 1.02, 0.1–7.3 Vs 0.58, 0.09–4.7, p = 0.25), Mcl-1 (1.16, 0.1–8.0 Vs 0.57, 0.05–3.7, p = 0.13) and Bax (1.23, 0.07–5.5 Vs 0.68, 0.08–3.9, p = 0.14) in AASV neutrophils than in HBD neutrophils; however, these differences were not statistically significant (Table 4). No significant correlation was observed between the rates of neutrophil apoptosis or necrosis in neutrophils from AASV patients and relative expression of pro-/anti-apoptotic factors. However, expression of Bcl-2A1 (Median, range: 1.96, 0.05–6.5 Vs 0.58, 0,09–4.7, p = 0.004) and Mcl-1 (1.49, 0.14–7.23, p = 0.007) was significantly higher in RA neutrophils than in HBD neutrophils (Table 4). Expression of pro and anti-apototic factors was not higher in neutrophils from PV patients and TP recipients than in HBD (Table 4).

**Table 4:** Gene expression of pro-/anti-apoptotic factors in neutrophils.

	HBD	AASV	PV	TP	RA
n	19	20	10	12	21
cIAP2-mRNA	0.93	1.1	1.06	0.77	1.14
Bcl2-A1-mRNA	0.58	1.02	0.55	0.76	1.96*
Bax-mRNA	0.68	1.23	0.49	0.55	0.77
Mcl-1-mRNA	0.57	1.16	0.38	0.68	1.49*

All results are expressed as median fold change relative to Cyclophilin A.

(*) P value<0.05, according to Mann-Whitney test and as compared to HBD.

HBD = healthy blood donors. AASV = ANCA-associated Systemic Vasculitis. PV = Polycythemia Vera. TP = renal transplant recipients. RA = Rheumatoid Arthritis.

Transcription factors involved in the process of granulopoiesis were quantified in neutrophils from HBD (n = 22), AASV (n = 25), RA (n = 10), PV (n = 10) patients and TP (n = 12). Results showed significantly higher mRNA encoding C/EBP-α and C/EBP-β in AASV patients than in healthy controls (Table 5). Neutrophils from PV patients had significantly lower levels of C/EBP-β and PU.1 than neutrophils from HBD. There was no significant correlation between mRNA levels of any of the transcription factors and the rate of neutrophil survival/apoptosis. On the other hand, there was a significant positive correlation between C/EBP-α and G-CSF levels in plasma (Spearman r = 0.7, p = 0.03, n = 9) among AASV patients.

**Table 5:** Gene expression of transcription factors in neutrophils.

	HBD	AASV	PV	TP	RA
n	22	25	10	11	10
C/EBP-α-mRNA	0.23	0.83**	0.08*	0.07	0.22
C/EBP-β-mRNA	0.35	3.35***	0.1**	0.2	0.75
PU-1-mRNA	0.85	1.5	0.3**	0.24	0.35

All results are expressed as median fold change relative to Cyclophilin A.

(*) P value<0.01,

(**) p value<0.001, and

(***) p value<0.0001, according to Mann-Whitney test and as compared to HBD.

HBD = healthy blood donors. AASV = ANCA-associated Systemic Vasculitis. PV = Polycythemia Vera. TP = renal transplant recipients. RA = Rheumatoid Arthritis.

Neutrophils from 31 AASV patients and 23 HBD had similar mRNA expression of survival/JAK2-pathway inhibitors (SHIP-1, SOCS1 and SOCS3; p>0.05). mRNA expression of growth factor receptors was analyzed in 19 AASV patients and 9 HBD; no significant differences were noted (G-CSFR, GM-CSFR alpha and beta; p>0.05).

## Discussion

Neutrophils release reactive oxygen species and proteases into the tissue microenvironment, leading to tissue inflammation and injury. When cultured *in vitro*, in the absence or presence of insufficient concentrations of neutrophil survival factors, these cells undergo spontaneous apoptosis [20]. Neutrophils are removed from tissues via necrosis or apoptosis, followed by phagocytosis by macrophages [21] [22]. Defects in apoptotic pathways could lead to the persistence of auto-reactive T- or B-cells and development of autoimmune disease, including AASV [23].

Our study is the first to demonstrate a lower rate of spontaneous apoptosis and longer *in vitro* survival in neutrophils from AASV patients as compared to neutrophils from HBD, SLE patients, and TP. Decreased apoptosis was also noted in neutrophils from PV and RA patients, in accordance with previously published data [24] [25]. An accelerated rate of apoptosis and decreased phagocytosis by macrophages for neutrophils from SLE patients has been reported previously [26]. Contrary to our results, Harper et al. showed that neutrophils from AASV patients, especially those with active disease, have an accelerated rate of apoptosis [12]. In this study involving 8 patients with active systemic vasculitis and 17 patients in remission, apoptosis was correlated with high mPR3 expression and high intracellular superoxide production. The neutrophils, however, were incubated in DMEM medium with 10% autologous serum, and in our study we did not use serum/plasma in the culture of neutrphils in AIM-V medium. They assessed neutrophil apoptosis after 12 and 18 hours while we assessed neutrophil apoptosis after 20 hours. Another difference is in the methods used to assess apoptosis. While we used simultaneous labeling of neutrophils by annexin-V and 7-AAD with subsequent measurement by FACS, Harper et al have used neutrophil morphology and fluorescence microscopy to define apoptotic neutrophils. In our study we had only two patients with active disease (BVAS>5), as we chose to study patients in remission in an attempt to understand early pathophysiological mechanisms and to exclude inflammation.

Delayed neutrophil apoptosis has been associated with several diseases and syndromes including sepsis, sleep apnea, cystic fibrosis, pneumonia, idiopathic pulmonary fibrosis, Behçet disease in the remission phase of uveitis, inflammatory bowel disease, systemic inflammatory response syndrome after major trauma and Kawasaki disease [27] [28] [29] [30]. The occurrence of reduced apoptosis in such a variety of disorders suggests a common underlying factor, such as chronic inflammation. However, we did not observe any correlation between rate of apoptosis and markers of inflammation or clinical parameters (CRP, BVAS score, GFR). Moreover, most of our AASV patients were having no or mild vasculitis activity as revealed by undetectable/low plasma levels of most of the cytokines tested in our study. Although the use of immunosuppressive drugs may have confounded the results, the difference between AASV and TP patients suggests that drugs could at most account for only a minor part of the prolonged neutrophil survival.

Increased proportion of CD177^+^/PR3^+^ subpopulation of neutrophils is seen in AASV, SLE, as well as in states associated with increased granulupoiesis such as sepsis. No correlation between rate of apoptosis and proportion of CD177^+^/PR3^+^ neutrophils was evident in our data. Growth factor signaling prolongs survival through production of anti-apoptotic factors and/or down regulation of pro-apoptotic factors. The expression of anti-apoptotic Bcl-2A1 is up-regulated by G-CSF, GM-CSF and LPS, which also promote neutrophil survival [31] [32], while Mcl-1 is up-regulated by GM-CSF, IL-1 and LPS [33]. c-IAP2, an Inhibitor of Apoptosis Protein (IAP), is selectively up-regulated by G-CSF, but not by GM-CSF [34]. The pro-apoptotic factor Bax is down-regulated in response to G-CSF, GM-CSF, IL-3 and IFN-γ [35]. In our study, the mRNA levels of these factors showed no correlation with reduced apoptosis or necrosis in neutrophils from AASV or PV patients. However, expression of Bcl-2A1 and Mcl-1 was significantly higher in neutrophils from RA patients than in HBD, Table 4.

Alteration in neutrophil growth factor signaling may cause abnormalities in apoptosis. We measured the expression of transcription factors involved in steady-state and emergency granulopoiesis [36] [37]. The mRNA levels of these factors (C/EBP-α, C/EBP-β and PU.1) were significantly higher in AASV patients than in HBD, but were normal in patients with RA or TP recipients. The target genes of these transcription factors include many important neutrophil proteins, including G-CSF receptor, GM-CSF receptor, myeloperoxidase, PR3, elastase, lysozyme and lactoferrin [38] [39]. Thus, elevated expression of these proteins in AASV neutrophils may enhance their susceptibility/sensitivity to cytokines. Also, the transcription factors may stimulate neutrophil survival and granulopoiesis directly, independent of G-CSF and GM-CSF and their respective receptors [40] [41]. Increase of c/EBP-β indicates ongoing granulopoiesis via the emergency pathway, which may lead to an increased amount of naïve neutrophils in the circulation with possibly an altered phenotype. Neutrophils from AASV patients had similar mRNA expression of survival/JAK2-pathway inhibitors and growth factor receptors as compared to HBD, suggesting that they may not be directly involved in reduction of apoptosis.

Plasma contains a multitude of neutrophil growth factors. Previous experiments measuring various cytokines (known to increase neutrophil survival) failed to show conclusive results. Christensson et al. showed that AASV patients, in remission, had higher circulating levels of soluble Fas than HBD and other disease controls [42]. sFAS is a circulating receptor of death signals in the plasma; its presence in plasma prevent interaction between FAS and FASl (Fas ligand), leading to prolonged survival [43]. We found significantly elevated sFAS levels in patients with AASV, suggesting a possible role in delaying apoptosis, although the biological significance of this finding is unclear. Elevated levels of sFAS could also be related to lymphocyte activation, which is already reported in AASV even during quiescent disease [44] [45]. G-CSF, GM-CSF and IL-3, enhance neutrophil survival and delay or prevent neutrophil apoptosis [46] [47] [48]. Plasma levels of IL-3 levels were normal in all vasculitis patient subgroups examined in this study. GM-CSF levels were higher than normal in 4 (of 44) AASV patients and in 8 (of 20) RA patients, Table 3. Interestingly, these 8 RA patients exhibited delayed neutrophil apoptosis as compared to the other RA patients, indicating different mechanisms for delayed apoptosis in RA and AASV. All other cytokines (TNF-α, IFN-γ, IL-1β, IL-2, Il-4, IL-6 and IL-8) tested in this study were undetectable in almost all plasma samples from healthy controls, and in most of the samples from AASV patients. This may be secondary to the fact that most of our patients were in complete remission.

Even though levels of G-CSF, GM-CSF and IL-3, were not elevated in AASV, they could still cause delayed apoptosis if AASV neutrophils were to have increased sensitivity to these cytokines. Our results show no difference between AASV patients and HBD regarding mRNA expression of G-CSF-Receptor (CD114) and GM-CSF-receptor (CD116). No previous studies on CD114 or CD116 expression have been published in regards to vasculitis patients, although studies on these receptors have been done in other inflammatory diseases such as inflammatory bowl disease and showed low/defective CD116 [49]. Future studies addressing these issues could be very interesting.

In summary, we find decreased spontaneous apoptosis in AASV, RA and PV, but mechanisms seem to differ. Our data demonstrating decreased apoptosis, increased mRNA levels of the C/EBP-α and C/EBP-β transcription factors, together with previous findings of increased proportion of double positive CD177^+^/PR3^+^ cells and increased transcription of the PR3 gene provides evidence for an altered neutrophil phenotype in AASV. A higher rate of neutropoiesis/granulopoiesis in AASV together with a lower rate of neutrophil apoptosis, may contribute to neutrophil accumulation in regions of inflammation. A definite mechanism for the delayed apoptosis in AASV could not be defined, our results are compatible with a role for an unidentified circulating factor, but intrinsic mechanisms cannot be ruled out. Novel survival pathways are emerging that have been reviewed recently by Witko-Sarsat et al [50], describe a cytosolic scaffold protein, Proliferating Cell Nuclear Antigen (PCNA), a cell cycle regulatory protein that is usually used by proliferating cells in proliferation, is used by neutrophils in regulating/inhibiting apoptosis. A recent study by Witko-Sarsat et al showed increased PCNA expression (both on mRNA and protein level) in neutrophils from AASV patients and that may give an explanation for our yet unexplained finding of prolonged neutruphil survival [51]. However, this postulate must be tested simultaneously in the same samples/neutrophils. Improved understanding of mechanisms by which neutrophil survival and apoptosis are regulated will help explain the pathophysiology of AASV and may have implications for the diagnosis and treatment, also of related diseases.
